# Intake, digestibility, and performance of lambs fed spineless cactus cv. Orelha de Elefante Mexicana

**DOI:** 10.5713/ajas.19.0328

**Published:** 2019-08-23

**Authors:** Levi Auto Lopes, Marcelo de Andrade Ferreira, Ângela Maria Vieira Batista, Michel do Vale Maciel, Rodrigo Barbosa de Andrade, Joana Albino Munhame, Tomás Guilherme Pereira da Silva, Daniel Barros Cardoso, Antonia Sherlânea Chaves Véras, Francisco Fernando Ramos de Carvalho

**Affiliations:** 1 Animal Science Department, Federal Rural University of Pernambuco, Dom Manoel de Medeiros street, s/n, Dois Irmãos, Recife, Pernambuco, 52171-900, Brazil; 2 Animal Science Department, Federal University of Amazonas, Macurany street, 1805, Jacareacanga, Parintins, Amazonas, 69152-420, Brazil; 3 Academic Unit of Garanhuns, Federal Rural University of Pernambuco, Av. Bom Pastor, 560, Boa Vista, Garanhuns, Pernambuco, 55292-270, Brazil

**Keywords:** Cactus, Cochineal, Ingestive Behavior, Performance, Ultrasound

## Abstract

**Objective:**

To evaluate the effects of the carmine cochineal-resistant spineless cactus genotypes cv. Orelha de Elefante Mexicana (*Opuntia*) and Miúda (*Nopalea*) on the intake and digestibility of nutrients, ingestive behavior, performance, and ultrasound measurements of growing lambs.

**Methods:**

Thirty-six male (non-castrated) Santa Inês lambs were used, with an average age of 6 months and an initial average weight of 22.0±2.9 kg. They were distributed in a completely randomized design with 3 treatments (Tifton hay, *Nopalea* and *Opuntia*) and 12 replications, using initial weight as a covariate. The experimental period was 86 days, with the first 30 days used for the adaptation of the animals to the facilities, diets and management, and the remaining 56 days used for evaluation and data collection.

**Results:**

The intake and apparent digestibility of dry matter (DM), organic matter (OM), crude protein (CP), neutral detergent fiber (NDF), total carbohydrates (TC), non-fibrous carbohydrates (NFC), and total digestible nutrients (TDN) showed a significant difference (p<0.05) as a function of the diets, with the *Nopalea* treatment (p<0.05) increasing DM intake (g/kg and % body weight [BW]), CP, TDN, and TC digestibility, whereas the Tifton hay diet led to a high (p<0.001) neutral detergent fiber corrected for ash and protein (NDFap) g/d intake, NDFap (BW %) and digestibility of said nutrient. There was no effect of treatments (p>0.05) on feeding time, however, rumination time and total chewing time were higher (p<0.05) for animals fed Tifton hay. The performance of the animals was similar (p>0.05). For the ultrasound measurements, *Nopalea* promoted an increase in the final loin eye area, compared to Tifton hay.

**Conclusion:**

The use of spineless cactus variety Miúda leads to the greater intake and digestibility of nutrients. The evaluated carmine cochineal-resistant spineless cactus genotypes are alternatives for semi-arid regions as they do not negatively affect the performance of growing lambs.

## INTRODUCTION

The use of alternative food resources, which are readily available in socially vulnerable regions, can be adopted as a means of alleviating food shortages, especially during times of deficit [1]. Thus, in recent years, there has been a great deal of research interest into the use of spineless cactus for animal feed, and its different species and genotypes for the important role they play in the success of sustainable agricultural systems in arid and semi-arid areas worldwide.

Spineless cactus is a good food alternative for sheep in semi-arid regions, which contributes to an increase in the productivity of these animals [2]. Cardoso et al [3] also report that the dietary inclusion of up to 450 g/kg (on a dry matter [DM] basis) of the Miúda spineless cactus improves the microbial efficiency, nutrient utilization and growth performance of lambs. According to Costa et al [4], increasing the spineless cactus levels in the sheep diet favors a high digestibility of the nutrients.

However, in recent years, the propagation of the carmine cochineal pest (*Dactylopius opuntiae*) has been a limiting factor for the cultivation of spineless cactus, mainly of the Redonda and Gigante varieties, directly affecting its production. The use of resistant genotypes has become a necessary strategy as an alternative to the utilization of spineless cactus in regions that are susceptible to this pest, with an emphasis on two cultivars: Miúda (*Nopalea cochenillifera* Salm Dyck) and Orelha de Elefante Mexicana (OEM, *Opuntia* spp.) [5].

Although some benefits of feeding small ruminants spine-less cactus are already known [1,4–6], responses to the use of carmine cochineal-resistant genotypes are still limited, especially for Orelha de Elefante Mexicana. The chemical composition and, therefore, the nutritive value of the spineless cactus, can be influenced by several factors, including the species and genotype [7]. Thus, the level of inclusion of this cactus in the diet, associations with different ingredients, and anatomical differences such as the presence of spines, among other factors, will have different nutritional implications and may directly or indirectly influence animal performance.

Given the above, it was hypothesized that lambs fed with spineless cactus OEM would present a nutritional and productive response similar to those fed with the spineless cactus variety Miúda, and superior to those fed with grass hay. Therefore, the objective of this study was to evaluate the effects of the use of spineless cactus Miúda and OEM on the nutrient intake and digestibility, ingestive behavior, performance and ultrasound measurements of growing lambs.

## MATERIALS AND METHODS

This research was conducted in the lamb’s sector of the Animal Science Department of the Federal Rural University of Pernambuco (UFRPE) in Recife, Brazil.

### Animals, management and sample collection

The animals were handled and cared for according to the guidelines and recommendations of the Animal Use Ethics Committee (CEUA) of UFRPE, under license number (142/ 2018). Thirty-six male (non-castrated) Santa Inês lambs were used, with an average age of 6 months and an initial average weight of 22.0±2.9 kg. The lambs were distributed into 3 treatments and 12 replications, in a completely randomized design, with the initial body weight (IBW) being used as the covariate. The experimental period was 86 days, with the first 30 days used for the adaptation of the animals to the facilities, diets and management, and the remaining 56 days used for evaluation and data collection. The experimental area was composed of individual bays (1.0×1.8 m), including drinkers and feeders, arranged in a covered shed. Before the start of the experiment, all the animals were identified, treated for the control of endoparasites with doramectin 1% (Dectomax, Guarulhos, SP, Brazil) at the dose of 200 mcg/kg of body weight (BW), and vaccinated against clostridiosis.

### Experimental diets

The diets were formulated to be isonitrogenated to meet the nutritional requirements of lambs weighing 25 kg, with an average daily gain (ADG) of approximately 200 g, according to the nutritional recommendations of the National Research Council [8]. Experimental diets consisted of three treatments: i) Tifton hay; ii) *Nopalea*, spineless cactus *cv.* Miúda (*Nopalea cochenillifera* Salm Dyck); and iii) *Opuntia*, spineless cactus *cv.* Orelha de Elefante Mexicana (*Opuntia* spp.). The ingredients and chemical composition of the experimental diets are presented in Tables 1, 2.

The spineless cactus was crushed daily, in a machine suitable for forage cactus processing. The hay was ground in a forage machine with an 8-mm sieve screen to reduce selection by the animals, and mixed with the other ingredients to be supplied as a complete feed.

### Chemical composition

Samples of the ingredients, leftovers, and feces were collected and pre-dried in a forced ventilation oven at 55°C for at least 72 hours, then milled with Willey-type knives, with 2.0 and 1.0 mm sieve screens, and analyzed according to Association of Official Analytical Chemists (AOAC) recommendations [9]. The DM (method 934.01), crude protein (CP, Kjeldahl N×6.25, method 981.10), ether extract (EE, method 920.39), organic matter (OM, method 930.05), ashes (method 942.05) and lignin (method 973.18) were analyzed. The neutral detergent fiber (NDF) concentration was analyzed using an ash-corrected, thermostable amylase based on the procedures described by Mertens [10] and Licitra et al [11], respectively, however, in this study, the samples were inserted into polyethylene vessels with 100 mL of neutral detergent and autoclaved [12]. The acid detergent fiber (ADF) content was determined according to Van Soest and Robertson [13]. The total carbohydrates content (TC) was calculated according to Sniffen et al [14] and the non-fibrous carbohydrates (NFC) content was determined according to Hall [15].

Non-protein nitrogen (NPN, fraction A) and neutral detergent insoluble proteins and acid detergent insoluble proteins (fraction C) were determined according to Licitra et al [11]. The true protein fraction (B) was sub-fractionated based on the ruminal degradation rates into B1 (rapidly degradable), B2 (degradable intermediate) and B3 fractions (slowly degradable) [14].

### Intake and digestibility of nutrients

To estimate voluntary intake, the leftovers were collected and weighed before each feeding. The intake was measured by the difference between the feed supply and the leftovers of each animal per day; the amount supplied was adjusted daily, based on the voluntary intake of the animal, with an estimated leftover of 15%. For the digestibility assay, fecal samples were collected for five consecutive days at alternate times (0, 2, 4, 6, and 8 hours), after the rations were provided, directly from the rectal ampulla. Samples were mixed per animal to form a composite sample for the period.

For the estimation of fecal dry matter production (FDMP), indigestible neutral detergent fiber (iNDF) was used as an indicator. Samples of 1.0 g of the concentrated feed and 0.5 g of hay, feces, and leftovers were incubated for 264 hours in the rumen of a fistulated buffaloe, according to the methodology described by Valente et al [16]. The remaining material from the incubation was subjected to neutral detergent extraction, and the residue was considered to be iNDF. The FDMP was estimated by the relation between the intake of the indicator and concentration in the feces.

For the estimation of total digestible nutrients (TDN), the equation described by Weiss [17] was used: TDN = (_D_CP+ _D_NDFap+_D_NFC+_D_EE×2.25), where: _D_CP, digestible crude protein; _D_NDFap, digestible neutral detergent fiber corrected for ashes and protein; _D_NFC, digestible non-fibrous carbohydrates; and _D_EE, digestible ether extract.

The DM degradability was estimated by the *in situ* technique using non-woven textile bags (100 g/m^2^), in which food samples were incubated (for 2, 4, 8, 12, 24, 48, and 72 h) in the rumen of a fistulated sheep. Data on the disappearance of DM were adjusted by a non-linear regression, which predicts the potential degradability (PD) of food using the model proposed by Mehrez and Ørskov [18]: PD = a+b (1–e^−ct^), where a, soluble fraction; b, potentially degradable fraction; and c, rate of degradation of fraction “b”. Effective degradability (ED) was calculated according to Ørskov and McDonald [19] as follows: ED = a+([b×c]/[c+k]), where k = estimated rate of passage of solids in the rumen.

### Feeding behavior

Observations of the ingestive behavior of animals were performed using the instantaneous scanning method proposed by Martin and Bateson [20]. The lambs were observed every 5 minutes for 24 hours each day for three consecutive days, starting immediately after the morning feed, totaling 72 hours of observation. The activities recorded for each lamb were rumination, feeding, and idling. The feed and rumination efficiencies (kg/h) of DM and NDF were calculated by dividing the intake of each of these nutrients by the total feeding time (feed efficiency) or rumination time (rumination efficiency).

### Performance and ultrasound measurements

The animals were weighed at the beginning and at the end of the experimental period to evaluate the total weight gain (TWG) and ADG. The TWG was obtained by the difference between the final body weight (FBW) and IBW: TWG = (FBW–IBW). The ADG was obtained through the relation between the TWG and the total days of the performance period until slaughter. Feed conversion (FC) was calculated by the relationship between DM intake and ADG.

At the beginning of the experimental and pre-slaughter period, the loin eye area (*Longissimus dorsi* muscle) and the fat thickness covering this muscle in the 12th and 13th rib were evaluated *in vivo* using an ultrasonic sweep (Pie Medical equipment, Aquila model with 3.5 MHz transducer, (Esaote Benelux, Maastricht, The Netherlands). The wool from the measuring area was removed by shearing. The transducer was placed between the 12th and 13th ribs, alongside the spine and parallel to the rib, to obtain measurements. The animals were manually immobilized and the acoustic gel was used for contact between the probe and the skin. All measurements were made on the left side 4 cm from the spine. The scanned images were measured using Imagej software to determine the initial loin eye area (ILEA), final loin eye area (FLEA), and subcutaneous fat thickness.

### Statistical analysis

The experimental design was completely randomized, with the initial weight of the animals as a covariate (continuous independent variables). The analyzed variables were interpreted using an analysis of variance, at a significance level of 5% as the critical probability level for type I error, using the Statistical Analysis System (SAS) version 9.0 with the general linear model procedure, according to the following model:

$$Y_{ij}=\mu +T_{i}+\beta (X_{ij}-X)+e_{ij}$$

Where *Y_ij_* = the observed dependent variable; *μ* = overall mean; *T_i_* = treatment effect (i = 1 to 3); *β*(*X_ij_* – *X*) = covariate effect (IBW); and *e_ij_* = experimental error. The means were compared by Tukey’s tests (p<0.05).

## RESULTS

### Nutrients in feeds

Diets with spineless cactus (*Nopalea* and *Opuntia*) presented low levels of DM, NDF, ADF, and high NFC in the chemical composition of diets, with little difference between them. The degradability of DM for the fractions “a”, “b” and ED diverged between treatments (Table 2).

### Intake and digestibility of nutrients

The intake and digestibility of DM, CP, OM, NDFap, TC, NFC, and TDN presented a significant difference (p<0.05) as a function of the diets (Table 3). The *Nopalea* treatment promoted higher (p<0.05) DM intake (% BW), CP, TDN, and TC digestibility, whereas Tifton hay led to higher (p<0.001) NDFap intake (g/d), NDFap (BW %) and the digestibility of said nutrient.

### Feeding behavior

There was no difference in treatments (p>0.05) for the feeding time, with mean values of 3.5 h/d (Table 4). However, rumination time and chewing time were higher (p<0.001) for the animals who received the treatments containing spineless cactus (*Nopalea* and *Opuntia*) than those who received the Tifton hay diet, but they did not differ between treatments containing spineless cactus. *Nopalea* provided higher (p<0.05) feed efficiency (g DM/h) compared to the Tifton hay diet, however, it did not differ (p>0.05) from that of lambs fed *Opuntia* compared to the other treatments. The rumination efficiency (g DM/h) was also lower for the animals fed the Tifton hay diet, compared to the other treatments.

### Performance

The performance of the animals was similar (p>0.05) between the varieties of spineless cactus (*Nopalea* and *Opuntia*) and the Tifton hay diet (Table 4), with averages of 36.0 kg for the final weight, 13.4 kg for the TWG, 240 g/d for the ADG and 5.34 for FC.

The *in vivo* measurements performed by ultrasonography were similar (p>0.05) for the ILEA, as expected, however, as a function of the experimental diets, *Nopalea* promoted an increase in the FLEA, in relation to Tifton hay (Table 4).

## DISCUSSION

In similar conditions to this study, Vieira et al [21] report that to maximize spineless cactus intake, a minimum of 150 g/kg DM of Tifton hay is required, coupled with an adequate amount of degradable protein. Nevertheless, according to Gebremariam et al [6], the use of spineless cactus for feeding sheep also causes abdominal distension. However, according to the data found for the degradability and rapid passage rate of spineless cactus diets (Table 2), the filling effect may be mild, and small breaks in intake may be sufficient to reduce ruminal and animal distension return to ingest the feed. It is worth mentioning that, in this case, the ingestion of DM may be related to the energy concentration and fermentation products. Thus, excessive amounts of nutrients may limit intake due to physiological regulation [22].

Rapisarda et al [23] reported the preference of lambs for foods that provide readily available energy, such as starch. This can be related to the high palatability of the *Nopalea* treatment, which presents a high carbohydrate content [24] and on average 205 g/kg DM starch [7]. This would probably explain the high intake of DM and TDN from this treatment. The lower NDFap intake of the spineless cactus treatments was due to the lower availability of this nutrient in the diet’s composition, as well as the difference between the genotypes, especially *Opuntia*.

Batista et al [7], who evaluated different genotypes of spine-less cactus, reported a high ruminal degradability of DM (701 g/kg), higher than the values found in the present study. Spineless cactus supplementation maximizes the ruminal fermentation capacity [25], as well as increases the synthesis of microbial protein and the production of volatile fatty acids [26–28]. However, the different carmine-resistant spineless cactus genotypes likely promote different ruminal fermentation patterns, considering the effects of the diets in terms of leading to the highest DM, CP, OM, NDF, and TC digestibility for the *Nopalea* treatment compared to *Opuntia*. It is possible that the fermentation pattern of this genotype in the lamb’s diet resulted in a better energy/protein balance, maximizing the availability and nutrient utilization for the ruminal microorganisms, consequently supplying volatile fatty acids and microbial protein to the animal.

According to the data related to the protein fractionation of different ingredients, although *Nopalea* presents higher participation of the indigestible fraction (C) compared to *Opuntia*, it also presents a higher content of NPN (A), of fast degradation. Together with the high rate of passage of both genotypes, part of the true protein (B), which is superior in *Opuntia*, is overpassed in the rumen, decreasing the retention and degradation time, and thus reducing the production of microbial protein and the digestibility of this nutrient. The same behavior was confirmed by Silva et al [5], who evaluated resistant spineless cactus genotypes (Miúda and OEM) for dairy cows and also reported higher CP and TDN intakes, and higher DM, OM, and CP digestibility for Miúda compared to OEM.

For the ingestive behavior presented by the animals, despite the similar feeding time between the treatments, the rumination time, as well as the total chewing time, was reduced with the diets with spineless cactus, compared to Tifton hay. Rumination rates are influenced by total NDF intake, which may be related to the retention of ruminal filling and content, which stimulates chewing, corroborating the previous facts.

The animals fed *Nopalea* showed a higher feeding efficiency (g DM/h, 66.7%) compared to Tifton hay; that is, they ate more DM per unit of time. Also, the increased rumination efficiency (g DM/h) for the spineless cactus treatments is related to the reduction of the effective fiber of the diets and is a reflection of the ruminal degradation rates and ruminal flow.

With the increase in nutrient consumption provided by the *Nopalea* treatment, we expected this to be reflected in the productive performance, however, considering the previously reported difference in ruminal filling and dietary rate, it is likely that a greater weight retention of abiotic content occurred in the gastrointestinal tract of the control treatment, which may have concealed the expected weight gain in the muscle and adipose tissues. Statistically, the animals gained similar amounts of absolute weight for each kilogram of DM consumed, regardless of whether they were fed Tifton hay, *Nopalea* or *Opuntia*.

Despite the animals presenting similar final weights, there was a greater indication of muscle development through the FLEA associated with the *Nopalea* treatment, before slaughter. There was a high positive correlation between the *in vivo* ultrasound measurements and the carcass composition [29], confirming the importance of this tool for carcass prediction.

## CONCLUSION

The use of spineless cactus variety Miúda leads to the greater intake and digestibility of nutrients. The evaluated carmine cochineal-resistant spineless cactus genotypes are alternatives to semi-arid regions as they do not negatively affect the performance of growing lambs.

## Figures and Tables

**Table 1:** Chemical composition of experimental diets ingredients (g/kg DM)

Items	Tifton hay	*Nopalea*	*Opuntia*	Soybean meal	Ground corn
Dry matter1)	895.5	123.6	97.2	882.7	877.1
Soluble fraction (a)	211.0	362.8	457.5	367.1	283.0
Insoluble fraction (b)2)	434.4	515.0	558.1	620.0	850.3
kd (%/h)3)	1.42	2.72	1.41	0.603	1.38
Effective degradation	307.1	544.3	580.3	433.8	466.9
Crude protein	86.0	40.0	55.0	487.0	85.0
Fraction A	25.1	23.4	15.0	17.7	15.3
Fraction B1+B2	26.7	42.1	58.4	76.6	70.1
Fraction B3	42.1	16.2	16.1	3.1	8.3
Fraction C	6.1	18.2	10.5	2.5	6.2
Ash	83.9	129.4	149.0	70.3	12.3
Organic matter	916.0	870.5	850.9	929.6	987.6
Ether extract	22.6	13.8	17.8	15.0	38.3
NDFap	669.4	252.7	198.0	134.5	146.7
Acid detergent fiber	336.1	137.1	95.3	116.7	24.4
iNDF	214.2	73.9	70.0	8.2	13.7
Lignin	66.7	22.7	24.4	11.1	5.1
Non-fibrous carbohydrates	138.0	563.9	580.0	293.0	717.6
Total carbohydrates	807.4	816.6	778.1	427.5	864.3

DM, dry matter; NDFap, neutral detergent fiber corrected for ash and protein; iNDF, indigestible neutral detergent fiber.

1) g/kg natural matter.

2) Potentially degradable insoluble fraction.

3) Rate of degradation of fraction b.

**Table 2:** Proportion and chemical composition of the experimental diets

Items	Treatments (g/kg)		

	Tifton hay	*Nopalea*	*Opuntia*
Ingredients (g/kg)			
Tifton hay	600	150	150
Miúda spineless cactus	0	450	0
OEM spineless cactus	0	0	450
Ground corn	270	271	273
Soybean meal	110	100	100
Ureia	5	14	12
Mineral mix1)	15	15	15
Total	1,000	1,000	1,000
Chemical composition (g/kg DM)			
Dry matter2)	890.8	234.8	190.3
Soluble fraction (a)	243.4	303.8	346.4
Insoluble fraction (b)3)	558.4	527.5	546.9
kd (%/h)4)	1.29	1.84	1.25
Effective degradation	358.6	445.5	455.6
Organic matter	924.0	904.2	895.3
Crude protein	142.1	141.8	143.2
Ether extract	25.6	21.5	23.4
NDFap	456.1	267.4	243.1
Acid detergent fiber	221.1	130.4	111.7
Non-fibrous carbohydrates	300.2	473.4	485.8
Total carbohydrates	756.3	740.8	728.8
Lignin	42.6	22.8	23.5
Total digestible nutrients	648.2	709.8	632.7

OEM, Orelha de Elefante Mexicana; DM, dry matter; NDFap, neutral detergent fiber corrected for ash and protein.

1) Nutrients/kg of product: calcium, 140 g, phosphorus, 70 g, magnesium, 1,320 mg, iron, 2,200 mg, cobalt, 140 mg, manganese, 3,690 mg, zinc, 4,700 mg, iodine, 61 mg, selenium, 45 mg, sulphur, 12 g, sodium, 148 g, and fluorine, 700 mg.

2) g/kg natural matter.

3) Potentially degradable insoluble fraction.

4) Rate of degradation of fraction b.

**Table 3:** Intake and digestibility of nutrients from lambs fed with spineless cactus genotypes resistant to carmine cochineal

Items	Treatments			SEM	p-value

	Tifton hay	*Nopalea*	*Opuntia*		
Intake (g/d)					
Dry matter	1,129±149^b^	1,290±175^a^	1,172±176^ab^	0.03	0.020
Organic matter	1,041±136^b^	1,168±155^a^	1,055±159^ab^	0.027	0.039
Crude protein	170±23^b^	192±24^a^	168±25^b^	0.004	0.010
NDFap	473±64^a^	331±44^b^	282±38^c^	0.016	<0.001
NFC	357±46^b^	609±85^a^	564±90^a^	0.022	<0.001
TDN	728±68^b^	916±130^a^	740±103^b^	0.023	0.001
Intake (% BW/d)					
Dry matter	3.92±0.19^b^	4.34±0.32^a^	3.94±0.34^b^	0.060	0.002
NDFap	1.64±0.08^a^	1.11±0.07^b^	0.95±0.6^c^	0.051	<0.001
Apparent digestibility (%)					
Dry matter	67.97±3.9^b^	75.14±3.2^a^	68.15±4.4^b^	0.87	0.001
Organic matter	70.01±4.1^b^	77.50±3.2^a^	71.17±4.0^b^	0.84	<0.001
Crude protein	71.50±4.0^ab^	72.91±4.2^a^	67.80±5.8^b^	0.87	0.038
NDFap	63.72±3.7^a^	58.74±2.8^b^	45.46±6.2^c^	1.51	<0.001
NFC	77.86±4.9^b^	86.49±3.8^a^	83.62±3.0^a^	0.89	<0.001

SEM, standard error of the mean; NDFap, neutral detergent fiber corrected for ash and protein; NFC, non-fibrous carbohydrates; TDN, total digestible nutrients; BW, body weight.

a–c The averages in the lines followed by different superscripts letters are statistically different by the Tukey test at 5% probability.

**Table 4:** Feeding behavioral variables, performance and ultrasound measurements of lambs fed with spineless cactus genotypes resistant to carmine cochineal

Items	Treatments			SEM	p-value

	Tifton hay	*Nopalea*	*Opuntia*		
Feeding behavior					
Feeding time (h/d)	4.20±1.25	3.19±1.23	3.10±0.79	0.210	0.072
Rumination time (h/d)	8.15±0.8^a^	6.29±1.5^b^	5.41±1.5^b^	0.301	0.001
Idle time (h/d)	11.65±1.6^b^	14.51±2.4^a^	15.48±2.0^a^	0.437	0.001
Chewing time (h/d)	12.34±1.6^a^	9.48±2.4^b^	8.51±2.0^b^	0.437	0.001
Feeding efficiency (g DM/h)	298±105^b^	497±185^a^	402±111^ab^	0.033	0.040
Feeding efficiency (g NDF/h)	125±45	126±49	97±26	0.014	0.240
Rumination efficiency (g DM/h)	141±33^b^	218±63^a^	241±102^a^	0.008	0.007
Rumination efficiency (g NDF/h)	59±13	56±16	58±23	0.003	0.905
Performance					
Initial body weight (kg)	22.4±3.1	22.6±3.3	22.8±2.2	-	-
Final body weight (kg)	35.0±3.0	36.7±5.0	36.5±4.1	0.704	0.491
Total weight gain (kg)	12.6±2.5	14.1±5.2	13.7±3.3	0.654	0.607
Average daily gain (g/d)	225±45	252±93	245±60	0.012	0.605
Feed conversion	5.28±1.4	5.68±1.8	5.07±1.3	0.265	0.512
Ultrasound measurements					
ILEA (cm^2^)	5.41±1.0	5.89±1.5	5.95±1.2	0.224	0.372
FLEA (cm^2^)	9.93±1.2^b^	12.46±2.8^a^	11.65±2.2^ab^	0.460	0.047
SFT (mm)	0.51±0.09	0.55±0.08	0.52±0.11	0.020	0.525

SEM, standard error of the mean; DM, dry matter; NDF, neutral detergent fiber; ILEA, initial loin eye area; FLEA, final loin eye area; SFT, subcutaneous fat thickness.

a,b The averages in the lines followed by different superscripts letters are statistically different by the Tukey test at 5% probability.
